# Heart Rate and Muscle Oxygenation Kinetics During Dynamic Constant Load Intermittent Breath-Holds

**DOI:** 10.3389/fphys.2021.712629

**Published:** 2021-07-22

**Authors:** Janne Bouten, Sander De Bock, Gil Bourgois, Sarah de Jager, Jasmien Dumortier, Jan Boone, Jan G. Bourgois

**Affiliations:** ^1^Department of Movement and Sports Sciences, Ghent University, Ghent, Belgium; ^2^Centre of Sports Medicine, Ghent University Hospital, Ghent, Belgium

**Keywords:** diving response, face immersion, dynamic apnea, near-infrared spectroscopy, bradycardia, peripheral oxygenation

## Abstract

**Introduction:** Acute apnea evokes bradycardia and peripheral vasoconstriction in order to conserve oxygen, which is more pronounced with face immersion. This response is contrary to the tachycardia and increased blood flow to muscle tissue related to the higher oxygen consumption during exercise. The aim of this study was to investigate cardiovascular and metabolic responses of dynamic dry apnea (DRA) and face immersed apnea (FIA).

**Methods:** Ten female volunteers (17.1 ± 0.6 years old) naive to breath-hold-related sports, performed a series of seven dynamic 30 s breath-holds while cycling at 25% of their peak power output. This was performed in two separate conditions in a randomized order: FIA (15°C) and DRA. Heart rate and muscle tissue oxygenation through near-infrared spectroscopy were continuously measured to determine oxygenated (m[O_2_Hb]) and deoxygenated hemoglobin concentration (m[HHb]) and tissue oxygenation index (mTOI). Capillary blood lactate was measured 1 min after the first, third, fifth, and seventh breath-hold.

**Results:** Average duration of the seven breath-holds did not differ between conditions (25.3 s ± 1.4 s, *p* = 0.231). The apnea-induced bradycardia was stronger with FIA (from 134 ± 4 to 85 ± 3 bpm) than DRA (from 134 ± 4 to 100 ± 5 bpm, *p* < 0.001). mTOI decreased significantly from 69.9 ± 0.9% to 63.0 ± 1.3% (*p* < 0.001) which is reflected in a steady decrease in m[O_2_Hb] (*p* < 0.001) and concomitant increase in m[HHb] (*p* = 0.001). However, this was similar in both conditions (0.121 < *p* < 0.542). Lactate was lower after the first apnea with FIA compared to DRA (*p* = 0.038), while no differences were observed in the other breath-holds.

**Conclusion:** Our data show strong decreases in heart rate and muscle tissue oxygenation during dynamic apneas. A stronger bradycardia was observed in FIA, while muscle oxygenation was not different, suggesting that FIA did not influence muscle oxygenation. An order of mechanisms was observed in which, after an initial tachycardia, heart rate starts to decrease after muscle tissue deoxygenation occurs, suggesting a role of peripheral vasoconstriction in the apnea-induced bradycardia. The apnea-induced increase in lactate was lower in FIA during the first apnea, probably caused by the stronger bradycardia.

## Introduction

Acute apnea or breath-holding is known to induce a series of physiological responses which are called the “diving response.” This response can be defined as a pattern of respiratory, cardiac, and vascular responses triggered by apnea (Gooden, 1994). The diving response is characterized by bradycardia, peripheral vasoconstriction, and an increase in blood pressure. It is believed to fulfill an oxygen conserving role (Schagatay et al., 1999; Foster and Sheel, 2005) prioritizing O_2_ delivery to the vital organs, such as the heart and the brain. Indeed, acute breath-holding is believed to reduce overall O_2_ demand (Gooden, 1994; Foster and Sheel, 2005; Costalat et al., 2017).

During exercise, however, the overall cardiovascular and metabolic demand increases, leading to an increased oxygen delivery to the working heart and skeletal muscles. During dynamic apnea, conflicting stimuli emerge simultaneously. At the cardiac level, the apnea-induced diving response elicits bradycardia through increased parasympathetic nerve stimulation, originating from the removal of the inspiratory-induced phasic tachycardia and reduced parasympathetic stimulation of lung stretch receptors (Bain et al., 2018). On the other hand, the exercise-induced sympathetic activity stimulates tachycardia to improve blood circulation to the working muscle. At the skeletal muscle level, increased muscle sympathetic nerve activity (MSNA) in response to apnea elicits peripheral vasoconstriction redistributing blood flow and prioritizing O_2_ delivery to the vital organs (Heistad et al., 1968; Leuenberger et al., 2001), while exercise evokes local peripheral vasodilation which increases muscle blood flow. As face immersion is known to further enhance the diving response (Kawakami et al., 1967; Schuitema and Holm, 1988), dynamic apnea performed in an aquatic environment, such as practiced in synchronized swimming, underwater hockey, and dynamic apnea disciplines, can be expected to enforce these conflicting responses (Bergman et al., 1972; Hurwitz and Furedy, 1986).

The diving response has been shown to overrule the cardiovascular responses to exercise during dynamic apnea reaching heart rate values around and below resting heart rate (Scholander et al., 1962; Asmussen and Kristiansson, 1968; Bergman et al., 1972; Butler and Woakes, 1987; Joulia et al., 2009; Ichinose et al., 2018). This is especially true when the face is immersed in cold water (Bergman et al., 1972; Andersson and Evaggelidis, 2009) and during light exercise, while the response is less apparent in more vigorous exercise (Asmussen and Kristiansson, 1968). A similar manifestation of the diving response overruling the cardiovascular response to exercise was observed for peripheral vasoconstriction. Leg blood flow decreases during dynamic dry apnea (DRA) reaching resting baseline values and as such, overriding the exercise induced response (Nishiyasu et al., 2012; Ichinose et al., 2018). This illustrates that peripheral vasoconstriction occurs not only in resting but also in exercising muscle tissue. The peripheral blood flow response also appears to be related to the magnitude of bradycardia as a more pronounced decrease in leg blood flow was seen in the group of subjects with the strongest decrease in heart rate (Nishiyasu et al., 2012).

The effect of limited peripheral blood flow on muscle tissue oxygenation has occasionally been studied in dry static apnea through NIRS measurements (Palada et al., 2007; Eichhorn et al., 2015, 2017; Ratmanova et al., 2016; Bouten et al., 2020). These studies show a steady, continuous decrease in muscle tissue oxygenation starting within 10s after apnea onset and further developing throughout the breath-hold. This illustrates that the body is successful in maintaining cerebral oxygenation, only falling below resting value in the last part of the static breath-hold (Eichhorn et al., 2015; Bouten et al., 2020). However, only two studies reported muscle tissue oxygenation during either short DRAs at 60% VO_2_max (Kume et al., 2013) and maximal dynamic face immersed apnea (FIA) at 30% of peak power output (Costalat et al., 2013). Although direct comparison is difficult due to different methodologies, dynamic FIA appears to evoke stronger muscle deoxygenation than dry static apnea. Data comparing muscle oxygenation for DRAs with FIAs are currently missing.

Lower O_2_ supply to the working muscle due to a decrease in heart rate and peripheral vasoconstriction can also be expected to alter metabolic pathways during exercise from aerobic to more anaerobic energy delivery. Indeed, lactate has been observed to increase throughout series of apneas, with a stronger response in dynamic breath-holds (Elia et al., 2021).

The aim of this study was to investigate cardiovascular and metabolic responses and their kinetics during short dynamic apnea and to examine the role of face immersion with cold water (15°C) in enhancing these responses. We hypothesized that (1) face immersion augments the bradycardia which develops throughout the breath-hold and (2) face immersion augments muscle tissue deoxygenation due to peripheral vasoconstriction. We also expect that (3) hemodynamic changes shortly before and at onset of apnea occur in a specific order. Additionally, we hypothesized that (4) apnea-induced increases in lactate concentration can be observed which are enforced with face immersion due to a more pronounced muscle deoxygenation.

## Materials and Methods

### Ethical Approval

The protocol was approved by the Ethics Committee of the Ghent University Hospital (EC UZG 2016/0809). After verbally clarifying test procedures and potential risks involved, written informed consent was signed by all subjects. For subjects who were still a minor at the onset of testing, a parent or guardian signed the informed consent as well. All participants underwent a medical screening by a skilled physician before engaging any tests.

### Population

Ten female subjects (17.1 ± 1.8 years old) volunteered to take part in this study. These subjects were unexperienced in apnea-related sports, but were engaged in physical training for 6.7 ± 4.2 h per week. All subjects were non-smokers and were declared to be in good general health. Subject characteristics can be seen in Table 1. All subjects were advised to avoid any vigorous activity 48 h prior to the test and instructed to eat and drink similarly and refrain from caffeinated and alcoholic beverages in the last 24 h before each test.

**Table 1:** Anthropometric and training characteristics, lung function parameters, and maximal incremental test parameters (mean ± SE).

General characteristics	
Age (years)	17.1 ± 0.6
Height (cm)	164.6 ± 1.7
Body mass (kg)	60.5 ± 1.6
Body fat (%)	19.5 ± 1.1
Max static apnea time (s)	118.2 ± 11.2
Exercise test results	
Ppeak (W)	265.9 ± 10.7
HRpeak (bpm)	192.4 ± 2.7
VEpeak (L.min^−1^)	86.0 ± 5.2
VO_2_peak (mL.min^−1^.kg^−1^)	40.2 ± 1.4
[La]peak (mmol.L^−1^)	11.6 ± 1.1
Lung function parameters	
FVC (L)	4.4 ± 0.2
FEV_1_ (L)	3.7 ± 0.2

*P, power; HR, heart rate; VE, ventilation; VO2, oxygen uptake; La, lactate; FVC, forced vital capacity; and FEV1, forced expiratory volume in 1 s.*

### Procedures

The study consisted of three different testing days and took place in the Sport Science Laboratory Jacques Rogge (Ghent University, Belgium) at a constant ambient air temperature of 18°C and humidity of 45%. All tests were performed at least 1 week apart to avoid residual effects of the previous test day. During the first day, all participants were subjected to a medical screening with ECG analysis, anthropometric assessment, and pulmonary function testing. During this test, heart rate (HR), muscle tissue oxygenation, and blood lactate were measured. Additionally, subjects performed a series of five maximal dry static seated apneas with 2-min rest intervals. After 30 min of rest, subjects performed a maximal ramp incremental exercise test on a cycle ergometer. The incremental test started with 3 min of cycling at 30 W, followed by a continuously increasing power output (30 W. min^−1^). During this test, maximal power output, pulmonary gas exchange, and lactate 30 s post-exercise were measured.

**Figure 1:**
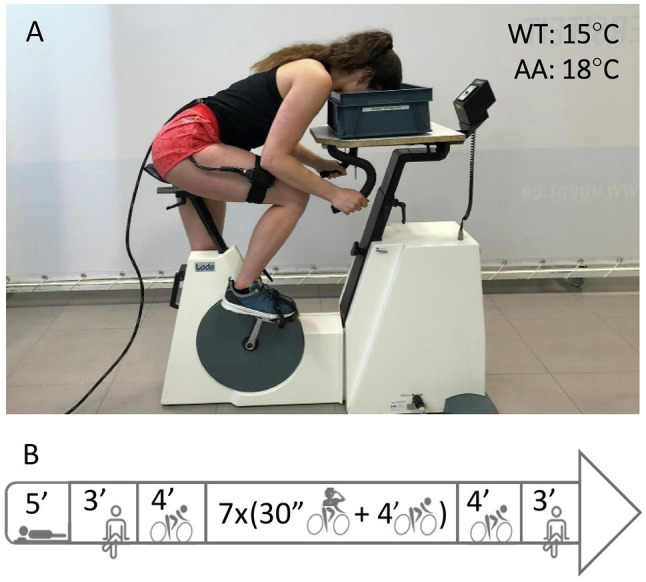
Panel **(A)** shows an example of the experimental setup. Ambient air (AA) was set at 18°C, water temperature (WT) in the container for face immersed apneas (FIA) at 15°C. Panel **(B)** shows an overview of the protocol starting with 5 min of supine rest, 3 min of BL measurements while seated on the bike followed by 4 min dynamic BL at 25% PPO. Then, subjects performed seven bouts of 30 s-dynamic apnea (AP) interspersed with 4-min cycling intervals, followed by a 4-min dynamic and 3-min seated recovery.

The following two test days, the subjects performed the two dynamic constant load intermittent apnea tests, one with face immersion (FIA) and one dry (DRA) in a randomized order. The protocol for the apnea tests is summarized in Figure 1. The test started with 5 min of supine rest in an isolated and silent room to measure the resting HR. After 3 min of seated rest in order to stabilize HR and NIRS data, subjects cycled 34.5 min at a cadence between 60 and 70 rpm and a power output of 25% of their peak power output obtained during the maximal incremental exercise test. During this period, subjects performed 7 bouts of 30-s dynamic apnea intervals, interspersed with 4-min bouts of regular cycling. The first bout was performed after 4 min of cycling. After the seventh (final) bout, subjects cycled for another 4 min followed by 3 min of seated rest on the ergometer to obtain recovery measurements. Capillary blood was collected for blood (lactate) determination 1 min after the first, third, fifth, and seventh apnea. Subjects were notified 30 s before each apnea and verbally guided with a 10-s countdown. Subjects were instructed to take a deep but not maximal breath. During apnea, subjects were not allowed to exhale and were verbally noticed if cadence was not kept constant. During the FIA test, a container with water (15°C) was placed on the steering wheel of the ergometer, positioned in a way that the subjects only had to bend the neck to submerge the face (Figure 1A). Subjects were instructed to submerge their face deeply, so the water contacted the skin in the regions surrounding the eyes, the forehead, the nose, and the mouth. After 30 s of apnea, the subjects lifted the head from the water and the face was dried with a towel. If the subjects were not able to sustain the 30-s apneas, breathing was resumed prematurely. During the 4-min breathing periods, the subjects’ head was positioned just above the water surface. This allowed minimal postural adjustments when starting or ending an apneic period. During DRA tests, the same procedure was repeated, but with an empty container placed on the ergometer’s steering wheel. All tests were performed at the same time of day for each participant to avoid bias by circadian rhythm.

### Equipment and Measurements

#### Anthropometric Assessment

Weight and height were measured using a SECA scale and measure (model 708, Seca GmbH, Hamburg, Germany). Ten skinfolds were taken to estimate the body fat percentage (cheek, chin, thorax 1, thorax 2, triceps, subscapular, abdomen, supra-iliac, thigh above the patella, and calf). All skinfolds were taken with a Harpenden skinfold caliper (Harpenden, West Sussex, United Kingdom) according to the ISAK (International Society for the Advancement of Kinanthropometry) guidelines. To avoid inter-observer variability, all skinfolds were taken by the same person (Fuller et al., 1991). Body fat percentage was calculated with the equation of Parizkova et al. (1961).

#### Incremental Exercise Test and Apnea Tests

All exercise tests were performed on an electrically braked cycling ergometer (Lode Excalibur Sport, Lode B.V., Groningen, Netherlands). A breath-by-breath gas analyzer (Jaeger Oxycon Pro, Viasys Healthcare GmbH, Höchberg, Germany) was used to perform lung function testing and measure gas exchange and ventilation during the incremental exercise test. Lactate was analyzed from a capillary blood sample collected from the right index finger (Radiometer ABL 90 Flex, Radiometer, Copenhagen, Denmark). Heart rate (HR) was recorded continuously (Polar HR monitor, Polar Electro, Kempele, Finland). After cleaning and shaving the skin, a near-infrared spectroscopy probe (NIRS; Oxiplex TS, ISS, Champaign, IL, United States) was positioned over the muscle belly of the M. Vastus Lateralis of the right quadriceps muscle and aligned with its vertical axis. The probe was securely attached with Velcro straps and tape. Muscle oxyhemoglobin (m[O_2_Hb]), deoxyhemoglobin (m[HHb]), and tissue oxygenation index (mTOI) were continuously derived based on the absorption of infrared light emitted at different wavelengths (692 and 834 nm) and averaged into 1-s bins.

### Data Analysis

Peak power output and peak HR were determined as the highest power output and HR reached during the incremental exercise test. The peak ventilation and peak oxygen uptake (VO_2_ peak) were defined as the highest 30-s average. The best single apneic performance out of the five static maximal apneas was set as the maximal static apnea time.

All time series (HR, m[O_2_Hb], m[HHb], and mTOI; 1 Hz) were synchronized. Baseline values for all four parameters were calculated for each apnea as the mean of the values between 90 and 30 s before the start of apnea. Extreme values represent the highest or lowest value obtained near or at the end of apnea, or in the first 30 s after apnea. The minimal value reached during or in the first 30 s after each apnea was taken for HR, mTOI, and m[O_2_Hb]. For m[HHb], the extreme value was defined as the maximal value achieved in the same time interval. Overshoot values represent the opposite extreme while normalizing but before returning to the actual baseline and were calculated as the maximal value obtained in the first 30 s following each apnea for HR, mTOI, and m[O_2_Hb]. For m[HHb], the minimal value was taken. Delta (∆) values for HR, mTOI, m[O_2_Hb], and m[HHb] were calculated as the difference between baseline and the extreme value for each apnea. For statistical analysis of the acute cardiovascular response, the average baseline values, values at −30, −25, −20, −15, −10, −5, 0, 5, 10, and 15 s after onset of apnea, extreme and overshoot values were calculated as the average of all seven apneas for each individual to obtain a general response for HR and NIRS values. Data at 20, 25, and 30 s are not presented because not all apneas were sustained for 20 s.

### Statistical Analysis

Statistical analyses were performed with IBM SPSS 26. Statistical significance was set at *p* < 0.05. Shapiro-Wilks tests were performed to check normality of the data. Sphericity was verified by Mauchly’s test of sphericity. When the assumption of sphericity was not met, the Greenhouse-Geisser correction was applied. Partial eta square ($\eta_{p}^{2}$) was used to indicate effect sizes for the RM MANOVA’s, while Cohen’s *d* was used to indicate effect sizes for pairwise comparisons. All data are presented as mean ± standard error.

#### Acute Cardiovascular Response

A 2 × 13 within-subjects RM MANOVA (Condition × Time Point) was used to analyze the acute response of the measures HR, mTOI, m[O_2_Hb], and m[HHb] over 13 time points (baseline, −30 s, −25 s, −20 s, −15 s, −10 s, −5 s, 0 s, 5 s 10 s, 15 s, extreme value, and overshoot value) for two conditions (FIA and DRA). Pairwise comparisons (least square differences) were used as *post-hoc* analysis. When a significant interaction effect was found, time points were one by one excluded from the analysis to determine when this difference started to establish.

#### Longitudinal Analysis

Three 2 × 7 within-subjects RM MANOVAs (Condition × Apnea number) were used to analyze the pattern of HR, mTOI, m[O_2_Hb], and m[HHb] for baseline, extreme, and ∆ values throughout the seven apneas (apnea number 1 through 7) for two conditions (FIA and DRA). Pairwise comparisons (least square differences) were used as *post-hoc* analysis.

A 2 × 4 within-subjects RM ANOVA (Condition × Lactate measurement) was used to analyze the evolution of lactate throughout the apnea tests for the measurements after the first, third, fifth, and seventh apnea for both conditions (FIA vs. DRA).

## Results

### Anthropometrics, Exercise, and Lung Function Tests

Subject characteristics are displayed in Table 1.

### Apnea Tests

#### General

Two out of 10 subjects completed all 14 dynamic 30-s apnea bouts (both face immersed apneas, FIA, and dry apneas, DRA). Two of out 10 subjects failed to complete any 30-s apnea. Subjects completed 28 of 70 DRA bouts and 29 of 70 FIA bouts without premature resumption of breathing. Incomplete apneas were sustained for 22 ± 1.4 s in both FIA and DRA, while total average apnea time for all apneas, complete and incomplete, was 25 ± 1.4 s in both conditions (*p* = 0.231).

#### Acute Cardiovascular Response

The 2 × 13 RM MANOVA (Condition × Time point) only showed a significant interaction effect for condition × time (*F* = 8.205, *p* < 0.001, $\eta_{p}^{2}$ = 0.477) for HR. Significant main effects were found for mTOI (*F* = 77.041, *p* < 0.001, $\eta_{p}^{2}$ = 0.895), m[O_2_Hb] (*F* = 36.421, *p* < 0.001, $\eta_{p}^{2}$ = 0.802), and m[HHb] (*F* = 34.409, *p* < 0.001, $\eta_{p}^{2}$ = 0.793) over time during acute apnea.

**Figure 2:**
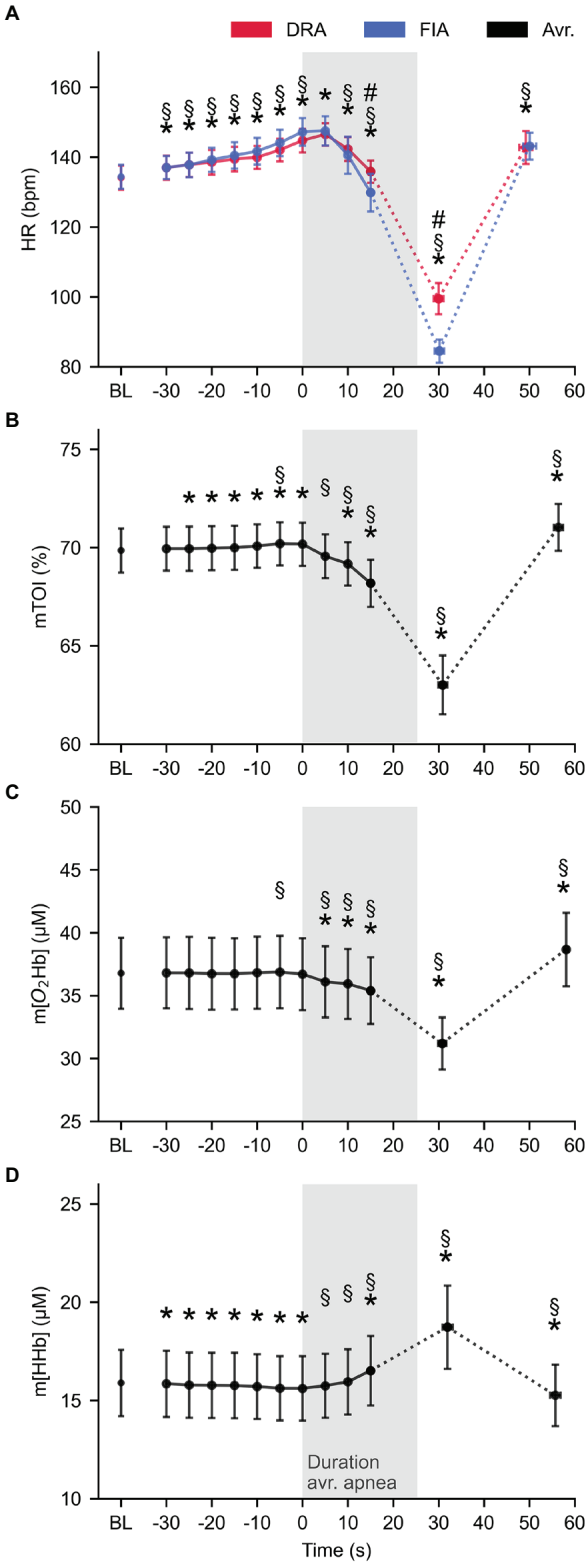
The average pattern for heart rate (HR, panel **A**), tissue oxygenation index (mTOI, panel **B**), oxygenated (m[O2Hb], panel **C**), and deoxygenated hemoglobin concentration (m[HHb], panel **D**) during apnea (*n* = 10). Blue lines represent FIA, red lines represent dynamic dry apnea (DRA), and black lines represent the average of both conditions. * = statistically different from BL at *p* < 0.05; § = statistically different from the previous value at *p* < 0.05; and # = statistical time × condition interaction effect at *p* < 0.05.

The interaction effect for HR revealed that the acute response in heart was more pronounced in FIA compared to DRA. This difference started to establish after 15 s (*F* = 10.104, *p* = 0.011, $\eta_{p}^{2}$ = 0.317) and was most obvious at the minimal HR (*F* = 15.391, *p* = 0.003, Cohen’s *d* = 1.20). Figure 2A shows that HR increased similarly from baseline to onset of apnea in both conditions (*F* = 3.156, *p* = 0.057, $\eta_{p}^{2}$ = 0.260), from 134 ± 4 bpm to 147 ± 4 bpm in FIA (*p* < 0.001, Cohen’s *d* = 1.09) and from 134 ± 4 bpm to 145 ± 3 bpm in DRA (*p* < 0.001, Cohen’s *d* = 0.98). HR reached a maximum of 148 ± 4 bpm in FIA and 147 ± 3 bpm in DRA 5 s after onset of apnea. From then on, HR started decreasing and did not reach values significantly below baseline in the first 15 s after onset of apnea (FIA: 130 ± 5 bpm, *p* = 0.273, Cohen’s *d* = 0.23; DRA: 136 ± 3 bpm, *p* = 0.495, Cohen’s *d* = −0.17). Minimal HR fell significantly below baseline in both FIA (85 ± 3 bpm, *p* < 0.001, Cohen’s *d* = 4.62) and DRA (100 ± 5 bpm, *p* < 0.001, Cohen’s *d* = 2.7) and was reached after 30 ± 1 s in both conditions which is around 5 s after termination of apnea.

mTOI slightly increased from baseline to onset of apnea (69.9 ± 0.9% at baseline to 70.2 ± 0.9% at T0, *p* = 0.003, Cohen’s *d* = −0.11) and immediately started decreasing after onset of apnea to 69.6 ± 0.9% at 5 s (*p* < 0.001, Cohen’s *d* = 0.21). mTOI reached values significantly below baseline after 10 s (69.2 ± 0.9%, *p* = 0.017, Cohen’s *d* = 0.20) and decreased even further till the minimum value (63.0 ± 1.3%, *p* < 0.001, Cohen’s *d* = 1.83) which was reached 30.5 ± 1.6 s after onset of apnea (Figure 2B). m[O_2_Hb] had already significantly decreased from 36.8 ± 2.7 μm at baseline to 36.1 ± 2.7 μm after 5 s (*p* < 0.001, Cohen’s *d* = 0.21). Figure 2C illustrates that m[O_2_Hb] steadily decreased (35.9 ± 2.7 μm after 10 s, *p* < 0.001, Cohen’s *d* = 0.23 and 35.4 ± 2.5.2 after 15 s, *p* = 0.001, Cohen’s *d* = 0.31) reaching an average minimum of 31.2 ± 1.9 μm (*p* < 0.001, Cohen’s *d* = 0.92) after 29.1 ± 2.0 s. m[HHb] decreased from 15.9 ± 1.6 μm at baseline to 15.6 ± 1.6 μm at the start of apnea (*p* < 0.001, Cohen’s *d* = 0.06) and immediately started increasing when the breath-hold started (*p* = 0.022, Cohen’s *d* = 0.04). m[HHb] only reached values significantly above baseline after 15 s (16.5 ± 1.7 μm, *p* < 0.001, Cohen’s *d* = 0.49). m[HHb] reached an average maximum of 18.7 ± 2.1 μm (*p* = 0.001, Cohen’s *d* = 0.13) after 32.0 ± 1.4 s (Figure 2D).

#### Longitudinal Analysis

The 2 × 7 RM MANOVA revealed that baseline values changed over time for all four parameters: Baseline HR (*F* = 8.414, *p* < 0.001, $\eta_{p}^{2}$ = 0.483), baseline mTOI (*F* = 41.282, *p* < 0.001, $\eta_{p}^{2}$ = 0.821), and baseline m[O_2_Hb] (*F* = 52.373, *p* < 0.001, $\eta_{p}^{2}$ = 0.853) increased throughout the series of apneas, while m[HHb] decreased (*F* = 17.594, *p* < 0.001, $\eta_{p}^{2}$ = 0.662). Similarly, the 2 × 7 RM MANOVA main effects for time revealed differences for extreme values over time for mTOI, m[O_2_Hb], and m[HHb]. Minimal HR following apnea tended to increase during the series (*F* = 2.566, *p* = 0.073, $\eta_{p}^{2}$ = 0.22). The lowest mTOI value (*F* = 33.830, *p* < 0.001, $\eta_{p}^{2}$ = 0.790) and m[O_2_Hb] (*F* = 24.068, *p* < 0.001, $\eta_{p}^{2}$ = 0.728) increased from the first to last apnea, while maximal values for m[HHb] decreased (*F* = 14.610, *p* < 0.001, $\eta_{p}^{2}$ = 0.619).

**Figure 3:**
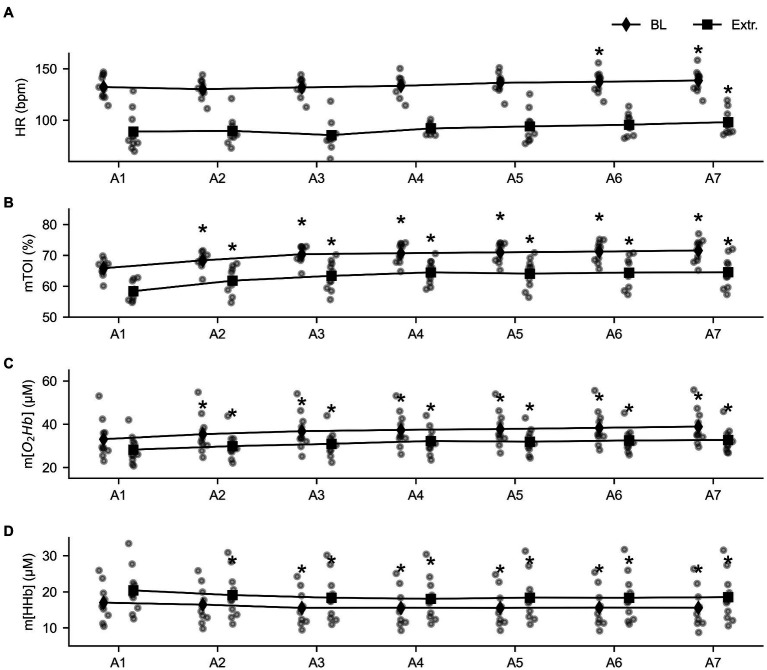
Overview of the evolution of the baseline (diamond shapes) and extreme values (squares) throughout the series of seven apneas for heart rate (HR, panel **A**), tissue oxygenation index (mTOI, panel **B**), oxygenated (m[O_2_Hb], panel **C**), and deoxygenated hemoglobin concentration (m[HHb], panel **D**). * = statistically different from A1 at *p* < 0.05.

Baseline and extreme values evolved similarly over the time course of the apneas (Figure 3), as observed by the non-significant main effect for the delta values (∆HR: *F* = 0.489, *p* = 0.814, $\eta_{p}^{2}$ = 0.052; ∆mTOI: *F* = 1.371, *p* = 0.274, $\eta_{p}^{2}$ = 0.132), ∆ m[O_2_Hb] (*F* = 2.358, *p* = 0.098, $\eta_{p}^{2}$ = 0.208), and ∆ m[HHb] (*F* = 2.292, *p* = 0.231, $\eta_{p}^{2}$ = 0.203).

**Figure 4:**
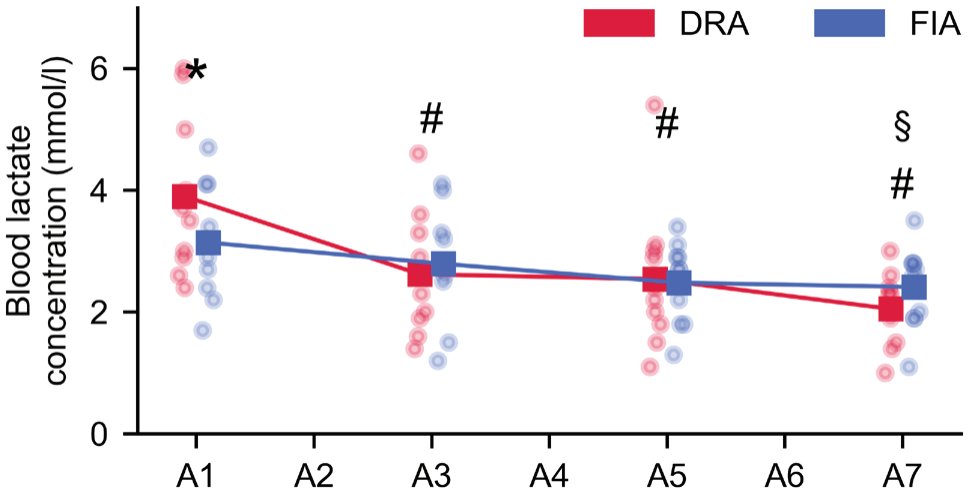
Evolution of lactate during the series of seven apneas (A1–A7 = measurement after the first–seventh apnea). The blue graph represents the average for FIA, while the red graph represents the average for DRA. * = significant difference between FIA and DRA at *p* < 0.05; § = significantly different from BL for FIA; and # = significantly different from BL for DRA.

Blood lactate values decreased over time from the value obtained 1 min after the first, to the value 1 min after the last apnea (*F* = 19.392, *p* < 0.001, $\eta_{p}^{2}$ = 0.683). This decrease was more pronounced for DRA than FIA (*F* = 4.386, *p* = 0.012, $\eta_{p}^{2}$ = 0.328; Figure 4); however, the blood lactate values at the respective time points only differed after the first breath-hold (*p* = 0.038, Cohen’s *d* = 0.57).

## Discussion

This study was the first to compare both heart rate and muscle oxygenation kinetics during constant load exercise with intermittent dynamic apneas between FIA and DRA. The main findings were that these types of dynamic apneas in subjects naive to breath-holds induced (1) a stronger bradycardia in FIA compared to DRA and (2) a significant decrease in mTOI which was similar in both conditions. Additionally, (3) an order of events was observed during which all muscle oxygenation patterns immediately started to decrease, while bradycardia occurred later, possibly suggesting that peripheral vasoconstriction can facilitate bradycardia. Last, (4) a higher lactate concentration following apnea was seen in DRA compared to FIA, although this difference was only observed after the first apnea.

Despite the short duration of apneas (the target time was 30 s, while actual time was on average 25 s), both FIA and DRA were successful in decreasing heart rate (HR) and muscle oxygenation. After an initial increase before and at onset of apnea, heart rate started to decrease after 5 s and did not fall below baseline in the first 15 s. Muscle oxygenation parameters changed immediately upon the onset of apnea. This is consistent with a quick onset of peripheral vasoconstriction and in line with the immediate increase in MSNA (Heusser et al., 2009) limiting blood delivery to the working muscle. As the muscle needs to maintain the same workload, O_2_ is still needed and muscle oxygenation therefore decreases.

Contrary to most studies comparing baseline values with end-apnea values and specific relative time points during breath-hold, we analyzed the data on an absolute time scale with short 5-s intervals. This allowed us to gain insight in the quick hemodynamic changes occurring before and at onset of breath-hold (Heusser et al., 2009) and especially in the order of events to understand the interaction of changes in heart rate and oxygenation. Looking deeper into the order of the response, it appears that the first response is an increase in heart rate, which already occurs before onset of apnea and lasts till 5 s after apnea. Aside from mental arousal and preparation, two mechanisms can be responsible for this increase in heart rate. First, the increase in lung stretch through deep inspiration stimulates the lung stretch receptors in the bronchi (Sroufe, 1971), which in their turn inhibit the cardiac vagal nerves and increase heart rate. Second, the deep inspiration at high lung volume is suggested to increase intrathoracic pressure which limits venous return and stroke volume and causes a drop in blood pressure and an increase in heart rate (Andersson and Schagatay, 1997; Schipke et al., 2019). This drop in blood pressure has been observed before in trained breath-hold divers (Schagatay et al., 1999; Sivieri et al., 2015; Ratmanova et al., 2016). Indeed, Heusser et al. (2009) showed that responses in blood pressure (MAP) and MSNA in the first 15–20 s of apnea closely resemble the responses of a Valsalva maneuver. This drop in blood pressure then increases peripheral resistance and leads to peripheral vasoconstriction, improving venous return. This quick vasoconstriction is consistent with our data showing an immediate decrease in m[O_2_Hb] and mTOI, and allows the heart rate to decrease and blood pressure to normalize. The order of mechanisms suggested above is consistent with our data in naive subjects showing that the onset of bradycardia is observed after the changes in muscle oxygenation, both in dynamic apnea (DRA and FIA) but also in dry static breath-hold (Bouten et al., 2020). These initial hemodynamic changes are mechanical and neural in nature and are therefore able to elicit the rapid changes observed in the short period before and immediately after onset of apnea. These initial reflexes are supported by chemical responses to hypoxia later in the apnea. For example, a chemoreflex-induced MSNA increase leads to sustained and/or increased peripheral vasoconstriction (Leuenberger et al., 2001; Heusser et al., 2009; Badrov et al., 2017). The origin of increasing MSNA during apnea is still debated and might be more related to the lack of ventilation than the chemoreflex (Badrov et al., 2017). However, the duration of breath-holds in this study is too short for these responses to elicit.

In accordance with previous studies for static apneas (Bergman et al., 1972; Andersson et al., 2002, 2004), the bradycardic response was more pronounced with FIA compared to DRA. This difference, however, only tended to manifest after 10 s and did not reach statistical significance in the first 15 s. Heart rate dropped significantly from 134 ± 3 bpm during cycling at baseline to a minimal value of 100 ± 5 bpm in DRA and to 85 ± 3 bpm in FIA. Our data suggest that stimulation of the trigeminal nerve does not alter the initial reflexes eliciting an increase in heart rate, but influences the pattern only when these initial reflexes, increasing heart rate, are overruled. Additionally, due to the practical setup, subjects immersed their face after deep preparatory inspirations and onset of apnea, causing a latency period between onset of apnea and face immersion. The response to face immersion therefore occurs later than the immediate respiratory-induced reflexes. Contrary, muscle oxygenation was similar in both conditions. This suggests that face immersion did not impact peripheral vasoconstriction in our study. A likely explanation would be that, while face immersion triggers the trigeminal nerve which regulates vagal activity and thus improves bradycardia (Lemaitre and Schaller, 2015), peripheral vasoconstriction results from sympathetic activation (Leuenberger et al., 2001) and would therefore be independent from face immersion. This is however in contrast with the observations of stronger increases in MSNA in short FIA compared to DRA (Fagius and Sundlöf, 1986) and greater increases in MAP during short dynamic FIA compared to DRA (Andersson et al., 2002), both supporting an enhanced sympathetic activation in response to face immersion (Heindl et al., 2004). This discrepancy in results might be related to differences in methodology. First, the short duration of the apneas in our study might be insufficient to develop a full dive response and differences might occur late in the apneas. However, increased MSNA (Fagius and Sundlöf, 1986) and increases in MAP and decreases in finger and forearm blood flow (Heistad et al., 1968) have been reported in apneas as short as 12 and 30s. Second, our subjects were inexperienced in breath-holding, while training is known to improve dive responses (Engan et al., 2013). A third consideration is that ambient air temperature (18°C) in our study was a lot lower than other studies and the difference between ambient air and water temperature (15°C) was only small. Indeed, Andersson et al. (2002) reported a water temperature of 10°C and ambient air temperature of 24°, while Fagius and Sundlöf (1986) showed the strongest increase in MSNA at the lowest water temperature (9°C) at an ambient temperature of 24°C. As cold air of 18°C already evokes noticeable increases in MSNA compared to 22°C (Fagius and Kay, 1991), it is possible that cold receptors stimulating MSNA were also partially stimulated without face immersion, making it more difficult to observe differences between FIA and DRA. A last consideration is that we only measured oxygenation in the working muscle. It is possible that perfusion to the working muscles is maintained to a certain level, while vasoconstriction appears to be more intense in inactive muscle tissues (Nishiyasu et al., 2012). Simultaneous oxygenation measurements of active and non-active muscles during dynamic apnea would be interesting to give more insight into this discussion.

When looking at the evolution of the baseline parameters during the prolonged constant load cycling at 25% of peak power output, we see that heart rate, mTOI, and m[O_2_Hb] gradually increase during the test. We hypothesize that this response can be explained by the interaction of the breath-holds, exercise, and thermoregulatory mechanisms, termed cardiovascular drift (Coyle and González-Alonso, 2001). Extreme values changed similarly to baseline values, indicating that the amplitude of the response did not change throughout the series of breath-holds. Indeed, this is reflected by the observation that all delta values were similar during the series, indicating that the response did not improve from the first to last apnea. This is probably due to the short nature of the apneas in this study. As the maximum of the breath-holds was set at 30 s, breath-hold time did not increase significantly throughout the series.

Blood lactate decreases during the series of apnea in both conditions. As blood lactate concentration is the net result of lactate production, diffusion to the blood, and elimination, a decrease is caused by either a lower lactate production during apnea, a higher lactate elimination rate during both apnea and cycling intervals, or a combination of both. As the magnitude of the response for both heart rate and muscle oxygenation is similar throughout the entire protocol, we do not expect lactate production to change. Improved lactate elimination on the other hand is a logical explanation as the continued exercise leads to better muscle oxygenation observed throughout the protocol, facilitating a better lactate clearance. Lactate concentration was significantly higher after the first apnea in DRA than FIA. The observation that peripheral oxygenation was similar during apnea in both conditions suggests similar local oxygen supply, while heart rate decreases less in the dry condition which could indicate that the overall metabolic demand reduces less in DRA. This might lead to a greater reliance on the anaerobic system to meet the demand in the dry apneas, leading to a higher lactate production, which might be reflected by a higher lactate concentration. However, this was only seen in the first apnea. Later in the protocol, when lactate elimination is expected to be enhanced due to prolonged exercise in the moderate intensity domain, the duration of the apneas is most likely too short to observe differences between the conditions.

## Conclusion

Our data show strong decreases in heart rate and muscle tissue oxygenation during short dynamic apneas in young female individuals naive to breath-holding. Contrary to previous research, only the response in heart rate was enforced through facial immersion, while muscle oxygenation was unaltered. This might indicate that the influence of face immersion on peripheral vasoconstriction is not a general response but only apparent in specific conditions and/or specific populations. Additionally, analyses of our data on a 5 s basis suggest an order of mechanisms through which heart rate increases due to inspiratory mechanical and neural mechanisms, followed by a quick onset of peripheral vasoconstriction as illustrated by rapid changes in muscle oxygen kinetics. Heart rate only starts to decrease after muscle tissue deoxygenation has started to establish, suggesting a role of peripheral vasoconstriction in the apnea induced bradycardia.

## Data Availability Statement

The raw data supporting the conclusions of this article will be made available by the authors, without undue reservation.

## Ethics Statement

The studies involving human participants were reviewed and approved by the Ethics Committee of the Ghent University Hospital. Written informed consent to participate in this study was provided by the participants’ legal guardian/next of kin.

## Author Contributions

All authors: conception, design, acquisition, analysis, and interpretation. JB and JGB: article drafting. All authors read, revised, and approved of the final version and agreed to be accountable for all aspects of the work.

### Conflict of Interest

The authors declare that the research was conducted in the absence of any commercial or financial relationships that could be construed as a potential conflict of interest.

## References

[ref1] Andersson J. P. A. Evaggelidis L. (2009). Arterial oxygen saturation and diving response during dynamic apneas in breath-hold divers. Scand. J. Med. Sci. Sports 19, 87–91. 10.1111/j.1600-0838.2008.00777.x, PMID: 18298614

[ref2] Andersson J. P. A. Linér M. H. Fredsted A. Schagatay E. K. A. (2004). Cardiovascular and respiratory responses to apneas with and without face immersion in exercising humans. J. Appl. Physiol. 96, 1005–1010. 10.1152/japplphysiol.01057.2002, PMID: 14578373

[ref3] Andersson J. P. A. Linér M. H. Rünow E. Schagatay E. K. A. (2002). Diving response and arterial oxygen saturation during apnea and exercise in breath-hold divers. J. Appl. Physiol. 93, 882–886. 10.1152/japplphysiol.00863.2001, PMID: 12183481

[ref4] Andersson J. Schagatay E. (1997). Effects of lung volume and involuntary breathing movements on the human diving response. Eur. J. Appl. Physiol. Occup. Physiol. 77, 19–24. 10.1007/s004210050294, PMID: 9459516

[ref5] Asmussen E. Kristiansson N.-G. (1968). The “diving bradycardia” in exercising man. Acta Physiol. Scand. 73, 527–535. 10.1111/j.1365-201X.1968.tb10892.x, PMID: 5708178

[ref6] Badrov M. B. Barak O. F. Mijacika T. Shoemaker L. N. Borrell L. J. Lojpur M.. (2017). Ventilation inhibits sympathetic action potential recruitment even during severe chemoreflex stress. J. Neurophysiol. 118, 2914–2924. 10.1152/jn.00381.2017, PMID: 28835525 PMC5686238

[ref7] Bain A. R. Drvis I. Dujic Z. MacLeod D. B. Ainslie P. N. (2018). Physiology of static breath holding in elite apneists. Exp. Physiol. 103, 635–651. 10.1113/EP086269, PMID: 29512224

[ref8] Bergman S. A. Campbell J. K. Wildenthal K. (1972). “Diving reflex” in man: its relation to isometric and dynamic exercise. J. Appl. Physiol. 33, 27–31. 10.1152/jappl.1972.33.1.27, PMID: 5037406

[ref9] Bouten J. Bourgois J. G. Boone J. (2020). Hold your breath: peripheral and cerebral oxygenation during dry static apnea. Eur. J. Appl. Physiol. 120, 2213–2222. 10.1007/s00421-020-04445-y, PMID: 32748010

[ref10] Butler P. J. Woakes A. J. (1987). Heart rate in humans during underwater swimming with and without breath-hold. Respir. Physiol. 69, 387–399. 10.1016/0034-5687(87)90091-0, PMID: 3659605

[ref11] Costalat G. Coquart J. Castres I. Joulia F. Sirost O. Clua E.. (2017). The oxygen-conserving potential of the diving response: a kinetic-based analysis. J. Sports Sci. 35, 678–687. 10.1080/02640414.2016.1183809, PMID: 27167834

[ref12] Costalat G. Coquart J. Castres I. Tourny C. Lemaitre F. (2013). Hemodynamic adjustments during breath-holding in trained divers. Eur. J. Appl. Physiol. 113, 2523–2529. 10.1007/s00421-013-2690-z, PMID: 23821240

[ref13] Coyle E. F. González-Alonso J. (2001). Cardiovascular drift during prolonged exercise: new perspectives. Exerc. Sport Sci. Rev. 29, 88–92. 10.1097/00003677-200104000-00009, PMID: 11337829

[ref14] Eichhorn L. Erdfelder F. Kessler F. Doerner J. Thudium M. O. Meyer R.. (2015). Evaluation of near-infrared spectroscopy under apnea-dependent hypoxia in humans. J. Clin. Monit. Comput. 29, 749–757. 10.1007/s10877-015-9662-2, PMID: 25649718

[ref15] Eichhorn L. Erdfelder F. Kessler F. Zur B. Hoffmann U. Ellerkmann R. K.. (2017). Influence of apnea-induced hypoxia on catecholamine release and cardiovascular dynamics. Int. J. Sports Med. 38, 85–91. 10.1055/s-0042-107351, PMID: 27454133

[ref16] Elia A. Barlow M. J. Wilson O. J. O’Hara J. P. (2021). Splenic responses to a series of repeated maximal static and dynamic apnoeas with whole-body immersion in water. Exp. Physiol. 106, 338–349. 10.1113/EP088404, PMID: 32421235

[ref17] Engan H. Richardson M. X. Lodin-Sundström A. van Beekvelt M. Schagatay E. (2013). Effects of two weeks of daily apnea training on diving response, spleen contraction, and erythropoiesis in novel subjects. Scand. J. Med. Sci. Sports 23, 340–348. 10.1111/j.1600-0838.2011.01391.x, PMID: 23802288

[ref18] Fagius J. Kay R. (1991). Low ambient temperature increases baroreflex-governed sympathetic outflow to muscle vessels in humans. Acta Physiol. Scand. 142, 201–209. 10.1111/j.1748-1716.1991.tb09148.x, PMID: 1877369

[ref19] Fagius J. Sundlöf G. (1986). Activity in muscle and skin nerve fascicles. J. Physiol. 377, 429–443. 10.1113/jphysiol.1986.sp016196, PMID: 3795097 PMC1182842

[ref20] Foster G. E. Sheel A. W. (2005). The human diving response, its function, and its control. Scand. J. Med. Sci. Sports 15, 3–12. 10.1111/j.1600-0838.2005.00440.x, PMID: 15679566

[ref21] Fuller N. J. Jebb S. A. Goldberg G. R. Pullicino E. Adams C. Cole T. J.. (1991). Inter-observer variability in the measurement of body composition. Eur. J. Clin. Nutr. 45, 43–49. PMID: 1855499

[ref22] Gooden B. A. (1994). Mechanism of the human diving response. Integr. Physiol. Behav. Sci. 29, 6–16. 10.1007/BF02691277, PMID: 8018553

[ref23] Heindl S. Struck J. Wellhöner P. Sayk F. Dodt C. (2004). Effect of facial cooling and cold air inhalation on sympathetic nerve activity in men. Respir. Physiol. Neurobiol. 142, 69–80. 10.1016/j.resp.2004.05.004, PMID: 15351305

[ref24] Heistad D. D. Abbound F. M. Eckstein J. W. (1968). Vasoconstrictor response to simulated diving in man. J. Appl. Physiol. 25, 542–549. 10.1152/jappl.1968.25.5.542, PMID: 5687360

[ref25] Heusser K. Dzamonja G. Tank J. Palada I. Valic Z. Bakovic D.. (2009). Cardiovascular regulation during apnea in elite divers. Hypertension 53, 719–724. 10.1161/HYPERTENSIONAHA.108.127530, PMID: 19255361

[ref26] Hurwitz B. E. Furedy J. J. (1986). The human dive reflex: an experimental, topographical and physiological analysis. Physiol. Behav. 36, 287–294. 10.1016/0031-9384(86)90018-1, PMID: 3961003

[ref27] Ichinose M. Matsumoto M. Fujii N. Yoshitake N. Nishiyasu T. (2018). Voluntary apnea during dynamic exercise activates the muscle metaboreflex in humans. Am. J. Physiol. Heart Circ. Physiol. 314, H434–H442. 10.1152/ajpheart.00367.2017, PMID: 29101169

[ref28] Joulia F. Lemaitre F. Fontanari P. Mille M. L. Barthelemy P. (2009). Circulatory effects of apnoea in elite breath-hold divers. Acta Physiol. 197, 75–82. 10.1111/j.1748-1716.2009.01982.x, PMID: 19254286

[ref29] Kawakami Y. Natelson B. H. DuBois A. R. (1967). Cardiovascular effects of face immersion and factors affecting diving reflex in man. J. Appl. Physiol. 23, 964–970. 10.1152/jappl.1967.23.6.964, PMID: 6065071

[ref30] Kume D. Akahoshi S. Song J. Yamagata T. Wakimoto T. Nagao M.. (2013). Intermittent breath holding during moderate bicycle exercise provokes consistent changes in muscle oxygenation and greater blood lactate response. J. Sport. Med. Phys. Heal. 53, 327–335. 23715258

[ref31] Lemaitre F. Schaller B. J. (2015). “The Trigeminocardiac reflex: a comparison with the diving reflex in humans,” in Trigeminocardiac Reflex. *Vol*. 11. eds. Chowdhury T. schaller B. J. (Elsevier), 193–206. 10.5114/aoms.2015.50974 PMC4424259 25995761

[ref32] Leuenberger U. A. Hardy J. C. Herr M. D. Gray K. S. Sinoway L. I. (2001). Hypoxia augments apnea-induced peripheral vasoconstriction in humans. J. Appl. Physiol. 90, 1516–1522. 10.1152/jappl.2001.90.4.1516, PMID: 11247954

[ref33] Nishiyasu T. Tsukamoto R. Kawai K. Hayashi K. Koga S. Ichinose M. (2012). Relationships between the extent of apnea-induced bradycardia and the vascular response in the arm and leg during dynamic two-legged knee extension exercise. Am. J. Physiol. Heart Circ. Physiol. 302, H864–H871. 10.1152/ajpheart.00413.2011, PMID: 22159992

[ref34] Palada I. Obad A. Bakovic D. Valic Z. Ivancev V. Dujic Z. (2007). Cerebral and peripheral hemodynamics and oxygenation during maximal dry breath-holds. Respir. Physiol. Neurobiol. 157, 374–381. 10.1016/j.resp.2007.02.002, PMID: 17363344

[ref8001] Parizkova J. (1961). Total body fat and skinfold thickness in children. Metabolism 10, 794–807. 14483890

[ref35] Ratmanova P. Semenyuk R. Popov D. Kuznetsov S. Zelenkova I. Napalkov D.. (2016). Prolonged dry apnoea: effects on brain activity and physiological functions in breath-hold divers and non-divers. Eur. J. Appl. Physiol. 116, 1367–1377. 10.1007/s00421-016-3390-2, PMID: 27188878

[ref36] Schagatay E. Van Kampen M. Andersson J. (1999). Effects of repeated apneas on apneic time and diving response in non-divers. Undersea Hyperb. Med. 26, 143–149. PMID: 10485514

[ref37] Schipke J. D. Lemaitre F. Cleveland S. Tetzlaff K. (2019). Effects of breath-hold deep diving on the pulmonary system. Respiration 97, 476–483. 10.1159/000495757, PMID: 30783070

[ref38] Scholander P. F. Hammell H. T. Lemessurier H. Hemmingsen E. Garey W. (1962). Circulatory adjustment in pearl divers. J. Appl. Physiol. 17, 184–190. 10.1152/jappl.1962.17.2.184, PMID: 13909130

[ref39] Schuitema K. Holm B. (1988). The role of different facial areas in eliciting human diving bradycardia. Acta Physiol. Scand. 132, 119–120. 10.1111/j.1748-1716.1988.tb08306.x, PMID: 3223302

[ref40] Sivieri A. Fagoni N. Bringard A. Capogrosso M. Perini R. Ferretti G. (2015). A beat-by-beat analysis of cardiovascular responses to dry resting and exercise apnoeas in elite divers. Eur. J. Appl. Physiol. 115, 119–128. 10.1007/s00421-014-2992-9, PMID: 25216993

[ref41] Sroufe L. A. (1971). Effects of depth and rate of breathing on heart rate and heart rate variability. Psychophysiology 8, 648–655. 10.1111/j.1469-8986.1971.tb00500.x, PMID: 5116829

